# What is the role of Ob&Gyn specialists in alleviating the declining fertility rates in the Nordic countries?

**DOI:** 10.1111/aogs.70033

**Published:** 2025-08-01

**Authors:** Rauni Klami, Antti Perheentupa

**Affiliations:** ^1^ Department of Obstetrics and Gynecology Turku University Hospital, University of Turku Turku Finland; ^2^ Ovumia Clinics Helsinki Finland

Low fertility has been apparent in several European countries for many decades. The Nordic countries have fallen below the level of replacement (i.e., total fertility index [TFI] of 2.1) already in the 1970s. However, this has not been reflected in the population numbers that have continued growing as a result of prolonged life expectancy as well as immigration. Alarmingly, the low total fertility index is quickly becoming a global trend rather than solely a phenomenon of the developed Western European countries. Currently, the TFI of half of all countries is below replacement value, and according to available data and models, the TFI of more than 90% of all countries will fall below 2.1 by the end of this century. The global population is estimated to peak at around 10 billion near the year 2100. Thereafter, a significant population decline is expected. There are also more dramatic forecasts, which suggest that the population peak will be reached by mid‐century and a substantial decrease will already be seen by the end of the century.^1^ At the moment, about 50% of all nations have a fertility rate below the replacement value of 2.1. Several countries are predicted to have a population decrease of >50% between 2017 and 2100. For clarity, it is important to understand that a TFI of 1.5 means that the number of children born will be halved in two generations.^2^

It is clear that in socioeconomically well‐developed countries, for example, the Nordic countries, far too many adults are unable to achieve the number of children that they would desire. According to the Family Federation of Finland, the Finns on average desire a family of two children; however, it is clear from the latest numbers that many families fail to reach this desired family size. Whereas the number of women and couples who choose voluntary childlessness may have increased, there is an evident gap in the number of children that couples wish to have and end up having.

There are multiple reasons behind the significant decrease in total fertility index. Social, economic, and educational factors are bringing the TFI down, particularly in advanced societies. Ecological conscience and fear of ecosystem collapse are sometimes given as reasons for choosing not to have children. Yet in a fairly recent evaluation in Sweden, 96% of high school students were planning to have children, girls slightly more than boys.^3^ The highly regarded importance of education and employment, particularly among women wishing for autonomy and independence, is causing a delay in establishing a family.

The rapid development of the “online lifestyle” is resulting in “dopamine culture” where instant gratification is expected. Research is showing that relationships have become shorter, and there are fewer marriages, more divorces, and consequently fewer opportunities to bring a child to an established partnership.^4^, ^5^ The dopamine culture appears to decrease human interactions, leading to less sexual activity overall.^6^ In addition, adult men report not being mature enough for parenthood as a reason for postponing or avoiding reproduction.^7^

Most of the socioeconomical changes can, at least in theory, be reversed with appropriate political and economic adjustments and policies. Finding effective solutions in society is, however, by no means an easy challenge. The Nordic countries are an excellent example of that. Despite the existence of fairly generous benefits in the form of parental leave, family allowance, and affordable childcare for the recent decades, the fertility rates in all of the Nordic countries are decreasing and are well below the replacement level. It is even possible that some of the benefits are detrimental in promoting starting a family at an early age. Earnings‐based family allowance may further promote late procreation; however, leaving some without children altogether.

We should also admit that we do not understand all of the underlying causes, particularly of voluntary childlessness, and further research is required into this serious issue so that at least some of the solutions may be evaluated and offered.

Immigration is currently alleviating this challenge to some degree; however, as the number of countries with a below replacement TFI value increases, the supply of immigrants will rapidly decrease. It is also important to acknowledge the brain drain and work force effect of emigration on the country of origin, as typically the skilled and educated individuals are welcomed elsewhere. Immigration is certainly not a long‐term solution to this, as the population growth is ceasing in developing countries as well.

It is clear that the level of knowledge on fertility awareness is fairly poor, and several significant gaps, particularly concerning the effect of age on fertility, are apparent.^8^ Basic knowledge and understanding among the public require improvement.

Health education on reproduction in schools needs to include sufficient basic information on fertility. The current curriculum stresses contraception and avoiding sexually transmitted diseases with minimal focus on fertility awareness. The effect of age as well as lifestyle choices of both men and women need to be neutrally presented. Female fertility decreases by about half by the age of 40 in comparison to the early 30s.^9^ By the age of 45, the likelihood of having a child as a consequence of a spontaneous pregnancy is approaching zero. This information needs to reach both boys and girls.

There are unrealistic expectations concerning the efficacy of infertility treatments. Social and traditional media headlines about motherhood in the late 40s relay unjustified and false information about female fertility as they typically do not mention that these pregnancies are a result of either using eggs frozen at an earlier age or, more commonly, donor eggs from a young donor. There are countries, for example, Japan, Greece, where 40% of IVF treatments are performed on women aged 40 years or more. In Europe, some 25% of IVF treatments are carried out in women of this age group.^10^, ^11^, ^12^ It is clear that the likelihood of ART leading to a live birth is drastically decreased with advancing maternal age. An increasing amount of data on the detrimental effect of advanced paternal age on fertility and reproductive health is becoming available.^13^ Access to fertility evaluation and treatment in the Nordic countries is estimated to be at a relatively good level.^14^ However, in order to carry out treatments efficiently, the patients need to apply for help without years of delay while the treatments have a good chance of being successful.^15^

Reproductive medicine specialists have a responsibility to offer high quality evaluation and treatment of infertility with the available resources. It is also important to inform the patients appropriately of the options and likelihood of success so that the number of women who drop out of fertility treatments remains minimal. However, all specialists in the field of obstetrics and gynecology should take part in ensuring that the patients they meet and treat have knowledge of the factors that affect fertility and the chances of having a successful pregnancy, that is, female age and lifestyle choices in particular. As establishing a family and having children is considered a basic human right, we should do our best that individual reproductive choices are based on appropriate information. We should make a habit of discussing fertility issues when evaluating the contraceptive alternatives with our patients.

The Nordic societies have changed, leading to a generation of young people with a set of goals and dreams different from their parents. Their views should be respected, while providing them with accurate information and encouraging them to think about fertility as a part of their future plans. We need to join forces to produce easily accessible, reliable online content related to fertility. It is necessary to train our generalist colleagues to address fertility issues when dealing with potentially harmful lifestyles and general disease. We should make sure that our schools teach fertility awareness, and this important message should be taken to our policymakers so that the appropriate action is taken to secure the future of the Nordic welfare societies.

## Data Availability

The data that support the findings of this study are available on request from the corresponding author. The data are not publicly available due to privacy or ethical restrictions.
